# Rituximab resistance in ITP and beyond

**DOI:** 10.3389/fimmu.2023.1215216

**Published:** 2023-07-28

**Authors:** Zhengrui Xiao, Irina Murakhovskaya

**Affiliations:** Division of Hematology, Department of Hematology-Oncology, Montefiore Medical Center, Albert Einstein College of Medicine, New York City, NY, United States

**Keywords:** ITP, immune thrombocytopenia, rituximab, rituximab refractory, rituximab resistance, novel therapies

## Abstract

The pathophysiology of immune thrombocytopenia (ITP) is complex and encompasses innate and adaptive immune responses, as well as megakaryocyte dysfunction. Rituximab is administered in relapsed cases and has the added benefit of inducing treatment-free remission in over 50% of patients. Nevertheless, the responses to this therapy are not long-lasting, and resistance development is frequent. B cells, T cells, and plasma cells play a role in developing resistance. To overcome this resistance, targeting these pathways through splenectomy and novel therapies that target FcγR pathway, FcRn, complement, B cells, plasma cells, and T cells can be useful. This review will summarize the pathogenetic mechanisms implicated in rituximab resistance and examine the potential therapeutic interventions to overcome it. This review will explore the efficacy of established therapies, as well as novel therapeutic approaches and agents currently in development.

## 1 Introduction

Immune thrombocytopenia (ITP) is a rare hematologic disorder with an incidence rate of 2.25 per 100,000 and slight female predomince (1). Diagnosis of ITP is based on isolated thrombocytopenia lower than 100×10^9^/L with the exclusion of other causes (2). Manifestations of ITP range from asymptomatic thrombocytopenia to severe life-threatening bleeding depending on the platelet level. In the recent update of the international consensus on the investigation and management of ITP, the management of ITP should commence when the platelet count drops below 20-30×10^9^/L to minimize the likelihood of bleeding. Other factors such as quality of life, existence of other hemostatic defects, impending surgeries, and susceptibility to trauma from occupation or lifestyle should also be considered to individualize therapy (2). Corticosteroids are the first-line therapy with an initial response rate of 75%, however 80% of patients eventually relapse or become corticosteroid-dependent (3). The second-line treatments are thrombopoietin receptor agonist (TPO-RA), splenectomy, rituximab and fostamatinib (3). Choosing between medications and agents should employ shared decision-making with the patient among the second-line treatment options. If the patient values treatment-free remission and avoidance of surgery, rituximab is the preferred treatment (4). However, the long-term response of rituximab is not sustained (5). In this review, we will discuss potential mechanisms of rituximab resistance in ITP, treatment strategies with available agents and new therapies in development which can potentially overcome the resistance.

## 2 Mechanism of ITP

While the cause of ITP was initially identified as a humoral factor by observing that the infusion of citrated whole blood or plasma from ITP patients into healthy recipients would result in a decrease in platelet count (6), more recently, the mechanism of ITP has been better understood. Circulating B cells and autoreactive plasma cells are responsible for the overproduction of autoantibodies (7, 8). Autoantibody binding with glycoproteins (GPs) IIb/IIIa, Ib, IV and Ia/IIa (9, 10) on the platelet surface is the crucial element in the pathogenesis of ITP. Autoantibody (singular) coated platelets are destroyed by a number of mechanisms. In the spleen, anti-GP IIb/IIIa autoantibody interacts with Fcγ receptors I and III on the macrophages to trigger phagocytosis (11) Platelet autoantibodies, particularly those targeting GP IIb/IIIa and Ib/IX (12), trigger the activation of the classical complement pathway, leading to the deposition and fixation of C1q and C4d on the platelet surface. This process facilitates the generation of the membrane attack complex (MAC), which leads to increased clearance of platelets (13, 14). Additionally, phagocytosis is also mediated by C3b interaction with macrophage complement receptor 1 (CR1) (15). Moreover, autoantibody is also observed to inhibit megakaryocyte maturation and production (16).

Desialylation of platelets is a physiologic mechanism responsible for removal of senescent platelets via hepatic Ashwell–Morell receptor (AMR), also stimulating TPO production and generation of new platelets (17). Murine models demonstrate that anti-GPIb/IX antibodies induce Fc-independent platelet activation and desialylation of platelets, which leads to platelet clearance via AMR (18). Patients with GpIb antibodies have been reported to have inferior response to Fc-dependent therapies such as corticosteroids and IVIG (19, 20), and multi-refractory patients with ITP have higher incidence of GPIbα antibodies and increased platelet activation and desialylation (21).

Apart from the humoral response, T cell dysregulation also plays a role in ITP pathogenesis. In ITP patients, it has been demonstrated that clones of CD4 positive T cells that can recognize GPIIb/IIIa are present in both the blood and spleen (22). Increase in CD4+ T helper 1 (Th1) cell activation (23, 24) and upregulation of Th17 cells (25) is associated with deficiency of CD4+CD25+ regulatory T cells (Treg), which are responsible for maintaining peripheral immune tolerance (26). CD3+CD8+ T cells are also increased in ITP patients and mediate cell-mediated cytotoxicity and cell-mediated platelet lysis (27). In addition, CD8+ T cells induce platelet desialylation leading to increase the clearance in the liver (28).

Megakaryocyte dysfunction also contributes to thrombocytopenia in ITP. Autoantibody IgG binding to the surface of megakaryocyte has been reported (29), associated with suppressed megakaryocyte production and impaired maturation (16) and decreased release of platelet from megakaryocyte (30). Autophagy is the process of eliminating old, damaged, or abnormal cytoplasmic proteins constituents and believed to be impaired in various of autoimmune disorders including ITP. The abnormal of autophagy in ITP can affect the differentiation of megakaryocytes into platelets (31). Disruption of megakaryocyte apoptosis by cytotoxic lymphocyte cytotoxicity also plays a role in megakaryocyte dysfunction leading to impaired platelet production (32, 33).

## 3 Rituximab in ITP

Rituximab, the first monoclonal antibody to be approved for therapeutic use, is a chimeric monoclonal antibody that targets CD20 (34). Rituximab’s Fab region has a specific binding affinity for the CD20+ target antigen. On the other hand, its Fc region can bind to various receptors, including the Fcγ receptors on immune effector cells, the neonatal Fc receptor, and the head of C1q (35). The binding and interaction leads to antigen-dependent cell-mediated cytotoxicity (ADCC), phagocytosis and complement-induced cell lysis (36). Due to the depletion of B cells, rituximab was used in various autoimmune disorders (37). Rituximab was first implemented in treatment of ITP around 2000 with responses, including complete, seen in patients refractory to steroids, splenectomy and other immune suppressants (38–40). Currently rituximab one of the recommended second-line therapies after steroid failure (2). Response to therapy is seen as soon as in the first week with median time to achieve response around 1 month (38, 41). Rituximab 375mg/m^2^ weekly for four doses is considered the standard dose based on the original clinical trial adopted from lymphoma therapy (38). In contrast, a low dose of 100mg/m^2^ weekly for four doses showed similar efficacy (42). Despite an initial response rate of nearly 60%, with half of the responses being complete, the long-term effectiveness of rituximab is not maintained. In fact, only 21% of adults and 26% of children were able to remain treatment-free based on a five-year outcome analysis (5). A number of predictive factors of response to rituximab has been evaluated. Several studies reported higher responses associated with female gender, younger age (40, 43), although this was not confirmed by other studies (5, 38, 44). Another study demonstrated that adolescent females with an ITP duration of less than 12 months had the longest response duration (45). In both pediatric and adult patients with ITP, the response to rituximab was not predicted by immunologic markers, such as antinuclear antibody, direct antiglobulin testing, immunoglobulin levels, and lymphocyte subsets, or primary versus secondary ITP (46). Although patients with anti-GPIIb/IIIa antibodies appear to have a higher response rate of 75% compared to those without at 46% (47), this finding is not sufficient to base treatment decisions regarding the clinical use of rituximab.

Resistance to rituximab in ITP patients can be mediated by a number of mechanisms.

## 4 Mechanism of rituximab resistance in ITP

### 4.1 B cell

As rituximab directly targets CD20+ B cells, the incomplete depletion of B cells has been linked to nonresponse to rituximab, and B cell reconstitution can predict relapse (48). During a 5-year follow-up study, patients who experienced a relapse had a faster reconstitution of B cells compared to those who maintained a response lasting over 2.5 years (5).

In a study evaluating B cell populations in splenectomy specimens of patients who did not respond to rituximab, two distinct groups of B cells were responsible for rituximab relapse. The germinal center B cell population was significantly expanded compared to the healthy donors along with increase in CD19+ naïve B cells, suggesting B cell reconstitution in the spleen after the clearance of rituximab. In addition, a population of preexisting mutated memory B cells characterized by up-regulation of several prosurvival genes and down-regulation of B-cell receptor (BCR) complex surface expression was identified, accounting for the escape of rituximab depletion (49). These groups of B cells can reactivate and differentiate into plasma cells once rituximab is cleared.

### 4.2 Plasma cell

Autoreactive anti-GpIIbIIIa antibody-secreting plasma cells have been observed in the patient’s plasma and bone marrow following the development of resistance to rituximab, suggesting their involvement in rituximab refractoriness (22). In a study analyzing the spleens of ITP patients, it was demonstrated that those who experienced rituximab failure had a greater number of autoreactive anti-GpIIbIIIa antibody-secreting long-lived plasma cells (LLPCs), despite near-complete B cell depletion in peripheral blood. Patients who responded to rituximab were also found to have lower levels of antibody-secreting plasma cells. Gene expression profiling of these plasma cells revealed a long-lived pattern characterized by overexpression of antiapoptotic genes while responders displayed a short-lived program (50). The results suggest that the long-lived plasma cells in the spleen could explain rituximab resistance. These pathogenic plasma cells have been demonstrated throughout the spleen, peripheral blood, and bone marrow, potentially leading to treatment failure (8). Higher concentration of B-cell-activating factor (BAFF), a member of the tumor necrosis factor family which promotes B-cell survival was detected in the supernatant of spleen cell cultures of rituximab refractory patients (50). In a subsequent study by the same group, using mouse model depletion of B cells led to elevated levels of BAFF and the appearance of splenic LLPCs. A combination of rituximab and BAFF-neutralizing antibody reduced the amount of LLPCs (51), suggesting that BAFF targeted therapy can potentially overcome the rituximab resistance by eliminating long-lived plasma cells.

### 4.3 T cell

In addition to its direct effect on B cells, rituximab also has an effect on the T cell population. As Treg cells are generally suppressed in ITP patients, infusion of rituximab has been shown to increase number and percentage of polyclonal Treg cells, thereby aiding in the suppression of Th1 cell function and decreasing inflammation (52). In rituximab non-responders, there was oligoclonal expansion of T cells with increased Th1/Th2 and Tc1 (IFN-γ single-positive CD8 cells)/Tc2 (IL-4 single-positive CD8 cells) ratio as well as the expression of Fas ligand on Th1 and Th2 cells (53). One potential reason for the lack of response to rituximab may be related to the specific role of CTLs in targeting and damaging platelets or megakaryocytes. This is supported by evidence indicating that rituximab non-responsive ITP patients exhibit an increase in splenic effector memory CTLs that produce large quantities of interferon-γ and display clonal restriction (54).

In a mouse model, CD4 positive T cells were also shown to play a role in extending plasma cell survival after rituximab treatment. Combination of CD4+ depleting therapy and anti-CD20 antibody led to a significant reduction in splenic plasma cells (51). Additionally, a separate study demonstrated that rituximab-resistant patients had significantly higher levels of CD4+CD45RO+ memory T lymphocytes in their peripheral blood compared to rituximab-responsive patients (55). T follicular helper cells (TFH) is a major T cell involving in B cell differentiation and proliferation in lymphoid organ. In chronic ITP patients who responded to treatment, the percentage of TFH is decreased significantly (56). Another study suggested that the follicular helper T cells can increase the level of BAFF in germinal center (57, 58).

## 5 Treatment to overcome rituximab resistance

### 5.1 Splenectomy

Historically, splenectomy as well as rituximab were utilized in second line therapy for ITP (2). There is no head to head comparison of the efficacy studies. The results from retrospective studies vary from similar efficacy (59) to longer response duration with splenectomy (60, 61) and a higher response rate with splenectomy (62). A review of 16 clinical studies suggested that rituximab carries a lower response rate and response duration than splenectomy (63) whereas meta-analysis in pediatric population suggested higher CR rates of 52% with 43% of responses persisting (64).

As discussed previously, pathogenic lymphocytes and plasma cells primarily residing in the spleen play a role in rituximab resistance. In that case, splenectomy could theoretically be the potential treatment for ITP beyond rituximab by eliminating the immune effector cells in the spleen. One retrospective study suggested that in rituximab non-responders who were treated with splenectomy the response rate was 100%. A retrospective study from Mayo Clinic found that patients receiving sequential splenectomy-rituximab or rituximab-splenectomy had similar 2-year freedom from relapse and were superior to those who received rituximab treatment alone (61).

### 5.2 FcγR signal transduction inhibition

The main mechanism of platelet destruction in ITP is phagocytosis of antibody coated platelets. FcγR signaling inhibition interferes with phagocytosis preventing platelet destruction. Syk and BTK are key tyrosine kinases that play a role in both B cell development and function as well as FcγR-mediated phagocytosis in macrophages (65–67). The neonatal crystallizable fragment receptor (FcRn) is involved in recycling of endogenous IgG including pathogenic autoantibodies (68). Agents targeting FcγR function present an alternative therapeutic approach in refractory ITP patients.

#### 5.2.1 Syk Inhibition

Fostamatinib, an oral potent and selective small molecule inhibitor of spleen tyrosine kinase (SyK), inhibits signal transduction of B-cell receptors and FcR-triggered Syk-dependent cytoskeletal rearrangement during phagocytosis, degranulation, and cytokine production. In the ITP model it been shown to decrease antibody-mediated platelet destruction (67). In two multicenter, double-blind, placebo-controlled, phase 3 clinical trials in patients with persistent/chronic ITP treated with fostamatinib 100mg twice a day with dose escalation to 150mg twice daily after 4 weeks in non-responders demonstrated overall response rate (ORR) of 43% compared to 14% on placebo (69), which was maintained in the long term follow up (70). Based on that, fostamatinib was approved by Food and Drug Administration (FDA) on April 2018 to treat ITP.

#### 5.2.2 BTK Inhibition

Bruton’s tyrosine kinase (BTK) is crucial for B cell development as well as cytokine and antibody production. Increased production of autoantibody associated with BTK expression is thought to be one of the mechanisms involved in the pathogenesis of systemic autoimmune diseases (65). In addition, BTK is also involved in FcγR-mediated phagocytosis in macrophages (71). The inhibition of BTK provides a potential target for autoimmune diseases including ITP. Rilzabrutinib, a covalent reversible BTK inhibitor, has demonstrated potent and durable inhibition of BTK. The reversible covalent inhibitors can maintain inhibition of a target proteins even after washout, potentially reducing off-target effects by minimizing drug exposure (72). BTK is homologous to Tec (tyrosine kinase expressed in hepatocellular carcinoma), which is expressed in platelets and plays a role in platelet aggregation through GPVI activation upon collagen stimulation. Rilzabrutinib is more selective for BTK than Tec compared to irreversible covalent BTK inhibitors, which is thought to result in fewer bleeding-related adverse effects (73). In a preclinical study, rilzabrutinib inhibited the activation and inflammatory activities of B cells and innate cells, reducing autoantibody mediated FcγR signaling (74). In a phase 1b/2 study of 60 heavily pre-treated including rituximab refractory ITP patients, rilzabrutinib demonstrated dose dependent improvement in platelet count with 40% of patients achieving the primary endpoint of platelet response (two consecutive platelet counts separated by ≥5 days of at least 50×10^3^/mm^3^ and an increase from baseline of at least 20×10^3^/mm^3^ without the use of rescue medication) at the highest dose of 400mg twice a day with median time to response of 12.5 days. Toxicity was low and no treatment-related adverse effect above grade 3 was observed (75). Currently rilzabrutinib 400mg twice daily is being evaluated in a phase 3 multicenter, randomized, double-blind clinical trial (NCT04562766).

### 5.3 FcRn inhibition

The neonatal crystallizable fragment receptor (FcRn) functions as intracellular shield from catabolism for immunoglobulin G (IgG), binding IgG and albumin in lysosomes under acidic conditions, protecting them from degradation, and recycling them to the cell surface (68). Blocking FcRn-IgG binding could facilitate lysosomal degradation of endogenous IgG, reducing the half-life of pathogenic IgG as the therapeutic target in antibody-mediated autoimmune diseases (76).

In a phase 2 clinical trial involving 66 patients with relapsed persistent or chronic ITP, the monoclonal anti-FcRn antibody Rozamolixizumab produced rapid and significant increases in platelet counts, along with substantial reductions in IgG levels, especially with single higher dose subcutaneous infusion (77). A phase 3 open-label extension study to investigate the long-term safety, efficacy and tolerability was recently completed (NCT04596995).

Efgartigimoid is a a human IgG1 antibody Fc-fragment with a high affinity for FcRn. In a randomized double blinded placebo controlled phase 2b trial of ITP patients refractory to previous lines of therapy four weekly IV infusions of efgartigimod induced a dose dependent rapid reduction of total IgG levels, which was associated with clinically relevant increases in platelet counts with almost half the patients achieving platelet count of ≥50 × 10^9^/L on at least two occasions, and a reduced proportion of patients with bleeding (78). In a multicenter, randomized, double-blinded, placebo-controlled trial in adults with persistent or chronic ITP recently reported (79), 51.2% of participants on efgartigimoid 10 mg IV weekly for 4 weeks then every 2 weeks achieved IWG response criteria versus 20% on placebo.

### 5.4 Sirolimus

Sirolimus is macrocyclic lactone with antifungal, antitumor and immunosuppressive activity and is a potent inhibitor of antigen-induced proliferation of T cells, B cells, and antibody production. Sirolimus complexes with family of intracellular binding proteins termed FKBPs (FK binding proteins) targeting mTOR and inhibiting the mTOR-mediated signal-transduction pathways, which results in cell cycle arrest in G1 phase (80). Activation of the mTOR protein is thought to play a significant role in the disruption of hematopoiesis in individuals with autoimmune disease (81). Sirolimus has been used successfully in children with autoimmune lymphoproliferative syndrome (ALPS) (82), as well as other primary or secondary autoimmune cytopenias (83), and has been particularly effective in managing autoimmune cytopenias in the setting of primary immunodeficiency and immune dysregulation. In a multicenter prospective study involving 30 children and young adults with relapsed/refractory autoimmune cytopenias, including 16 patients previously treated with rituximab, all 12 children with ALPS achieved a lasting complete response (CR). Additionally, CRs were observed in 8 out of 12 patients with multilineage cytopenias associated with common variable immunodeficiency, Evans syndrome, or systemic lupus erythematosus (84). In a preliminary report of a prospective multi-center clinical trial in patients with ITP, 66 patients who failed the second-line therapy, including 30% who received rituximab, 46/66 (70%) responded to sirolimus 2mg orally every day at 3 months with 45% responses being complete (85). In a longer follow-up, the overall response rate (ORR) was 70% and 65% at 6 months and 12 months, respectively. Responders demonstrated a decrease in the proportion of Th2 and Th17 cells, along with an increase in the percentage of M-MDSCs and Tregs, suggesting that sirolimus could potentially restore peripheral tolerance (86). In a prospective randomized observation trial of 43 patients with chronic ITP who relapsed after multiple prior therapies including rituximab, combination of low dose prednisone with sirolimus was compared to cyclosporine and sirolimus. Although ORR was similar (58% versus 62%), treatment with sirolimus and prednisone was associated with a higher rate of sustained response (68% versus 39%, P < 0.05) and a increase in Treg cell levesl (87).

### 5.5 Plasma cell inhibition

Given role of plasma cells in ITP relapse, plasma cell inhibition is a therapeutic target, particularly in rituximab relapsed patients.

#### 5.5.1 Proteasome inhibition

Bortezomib, first proteasome inhibitor approved for treating multiple myeloma, inhibits the ubiquitin-proteasome proteolytic pathway responsible for intracellular protein turnover, disrupting the cell cycle, inducing apoptosis, altering the bone marrow microenvironment and inhibiting nuclear factor kappa B to cause plasma cell depletion (88). By inducing apoptosis in antibody-secreting cells such as plasma cells and memory B cells, bortezomib leads to a reduction in antibody secretion (89, 90). Bortezomib also has been shown to have both immunosuppressive and immunostimulatory effects which is widely used in immune mediated disorders. It reduces the number of CD4 T cells and decreases their production of Th1 cytokines while also increasing the population of regulatory T cells (Tregs) (88). Bortezomib has been successfully used in several antibody mediated disorders, including thrombotic cytopenic purpura, warm autoimmune hemolytic anemia, and cold agglutinin disease (91–94). Bortezomib use in ITP treatment is limited in case reports. Therapy was successful in three out of four cases reported in the literature (95–98). However, use of additional immunosuppressive therapies could have a confounding effect on response to bortezomib. Bortezomib is currently being evaluated in refractory ITP, alone and in combination with rituximab in two clinical trials (NCT05599880, NCT03443570).

#### 5.5.2 CD38 inhibition

CD38 is a membrane glycoprotein which is present in hematopoietic cells, including plasma cells which is involved in cell adhesion, migration, and signal transduction. Daratumumab is a high-affinity IgG1 monoclonal antibody against a unique CD38 epitope, it clears CD38-positive plasma cells via antibody-dependent cellular cytotoxicity and complement-dependent cytotoxicity (99). Due to the plasma depletion effect, daratumumab has been used in autoimmune hemolytic anemia and lupus (100–103). In case reports and case series, daratumumab has shown efficacy in the context of post-allogeneic bone marrow transplant associated autoimmune thrombocytopenia (104–106) as well as other causes of secondary ITP (107, 108) and most recently in a relapsed multi refractory primary ITP (109, 110). A phase 2 clinical trial is currently evaluating the efficacy and safety of daratumumab in patients with ITP who did not respond to at least two prior therapies (NCT04703621). During the safety run-in, two out of three enrolled patients responded to the treatment at week 12, with one patient experiencing a relapse by week 24 (111). Another anti-CD38 monoclonal antibody, mezagitamab (TAK-079), a fully human IgG1 is currently being studied in chronic and persistent ITP (NCT04278924).

### 5.6 B cell inhibition

#### 5.6.1 BAFF inhibition

Given the role of BAFF in failure of B cell depleting therapy with rituximab in ITP, BAFF inhibition is emerging as an important therapeutic target to mitigate rituximab resistance. Belimumab is a human IgG1λ monoclonal antibody directed against BAFF which causes reversibly decrease of B lymphocyte production (112). Belimumab already shows its efficacy in autoimmune disorders including lupus (113). Based on the assumption of the synergistic effect of anti-CD20 and anti-BAFF, belimumab was administered in conjunction with rituximab in a phase 2b trial in 15 non-splenectomized patients with chronic and persistent ITP who failed at least one prior line of therapy. Patients received two doses of rituximab 1000mg 2 weeks apart combined with 5 infusions of belimumab 10mg/kg at weeks 0, 2, 4, 8 and 12. Overall response was 80% at 1 year follow up, including 66.7% complete responses. The combination arm resulted in similar B cell repopulation compared to patients who received rituximab alone, but there was a significant decrease in T-follicular helper cells (114). Addition of subcutaneous belimumab to rituximab is currently investigated in a randomized placebo-controlled phase 3 trial (NCT05338190).

Ianalumab is an afucosylated humanized IgG1 monoclonal antibody directed against the BAFF receptor. In addition to BAFF receptor blockade that interrupts BAFF-mediated important signaling for B-cell maturation, proliferation, and survival, ianalumab induces direct lysis of B cells by antibody-dependent cellular cytotoxicity, resulting in a rapid and profound B cell depletion of long-lasting duration (115–117). The efficacy and safety of lanalumab in addition to standard of care for first line and second line treatment of ITP are currently evaluated by phase 3 trials (NCT05653349, NCT05653219).

#### 5.6.2 CD19 directed therapy

CD19 is expressed on B cells as well as plasma cells. Inebilizumab is a humanized anti-CD19 monoclonal antibody that targets and depletes CD19-expressing cells via antibody-dependent cell-mediated cytotoxicity. It is currently approved for treatment of neuromyelitis optica spectrum disorder (118) and is being evaluated in other antibody mediated diseases including IgG4-related Disease (NCT04540497), myasthenia gravis (NCT04524273), systemic sclerosis (NCT05198557) and autoimmune encephalitis (NCT04372615). Hypogammaglobinemia with associated infectious complications have been reported in 20% of patients along with 12% risk of infusion reactions (118).

Obexelimab (XmAb5871) is an anti-CD19 non-depleting B cell monoclonal antibody that co-engages B cell antigen receptor complex and Fcγ receptor IIb inhibitory receptor resulting in inhibition of B cell proliferation, antibody secretion and plasma cell differentiation (119, 120) and is currently being investigated in SLE (NCT02725515) and IgG4-related disease (NCT05662241).

CD19 inhibition can be a potential therapeutic targets in patients who fail to respond to rituximab due to reconstitution of CD19+ B cells (49).

### 5.7 T cell inhibition

#### 5.7.1 Decitabine

Decitabine, a DNA methylation inhibitor with antimetabolite effect approved in myeloid hematologic disorders which induces hypomethylation when incorporated into DNA (121). In a preclinical study, low-dose decitabine increased the Treg cells and enhanced their immunosuppressive function, decreased the Th1 and Th17 cells and proinflammatory cytokines and inhibited STAT3 activation (122). Low dose decitabine has also demonstrated to restore the methylation level and expression of the programmed cell death protein 1 (PD-1) promoter, activating PD-1 signaling pathway and resulting in decreased number and cytotoxicity of CD8+ T cells in ITP patients (123). A multi-center prospective study evaluated low dose of decitabine 3.5mg/m^2^ for 3 consecutive days every 4 weeks for 3 cycles in 45 refractory ITP patients, 23 were previously treated with rituximab. The ORR was 51% (17.8% CR) at the end of therapy and 31% at 12 months with responses seen on retreatment (124).

#### 5.7.2 Mycophenolate mofetil

Mycophenolate mofetil (MMF) is a prodrug of mycophenolic acid which is an inhibitor of inosine-5-monophosphate dehydrogenase, which leads to l depletion of guanosine nucleotides in T and B lymphocytes, inhibition of proliferation, suppression of antibody production and cell-mediated immune response (125). In patients with ITP treatment with MMF was associated with changed in T cell lymphocytes, decreased inflammatory markers (126), decreased cytotoxic CD8+ T cells (127), suggesting that MMF works via T cell regulation. Among the case reports and case series, MMF is effective either as a single agent (128–130) or combined with other agents (131) in relapsed/refractory ITP. MMF was previously widely used as the second-line therapy in pre-rituximab era in Europe and Asia. Several clinical studies suggest the efficacy and effectiveness of MMF after steroids and splenectomy (127, 129, 132–136), with ORR ranging 38.9%-80%, although platelet response can take 4 to 6 weeks. MMF also demonstrated efficacy after rituximab therapy. In a retrospective study of 46 patients with severe ITP, all requiring second-line therapy, approximately one-third of them having received rituximab, the ORR was 52% with 33% of patients achieving complete response. There was no difference in response rates between patients who had previously received rituximab and those who had not (135). MMF has been evaluated in the first-line setting in a multi-center, open-label, randomized trial in United Kingdom as addition to corticosteroids in the first-line. Compared to steroids alone, patients who received steroids plus MMF achieved superior treatment response and experienced less treatment failure, albeit at the cost of reporting lower quality-of-life outcomes (136).

#### 5.7.3 Cyclosporin

Cyclosporin is an immunosuppressant that inhibits the phosphatase activity of calcineurin regulating gene expression in activated T cells and blocking signaling pathways triggered by antigen recognition (137). Cyclosporin was used sporadically pre-rituximab era as second-line therapy after failure of steroids and splenectomy, with evidence level limited to case reports, retrospective case series and one small prospective study (138–143). The ORR ranged from 55% to 100% with majority of responses sustained. A four week course of high-dose dexamethasone, low-dose rituximab 100mg on days 7, 14, 21 and 28 plus oral cyclosporin 2.5 to 3mg/kg daily was prospectively evaluated in a single-arm phase 2b study of 20 patients most of whom received at least three lines of therapy. Although overall response rate was 60%, relapse-free survival at 12 and 24 months was 92% and 76% respectively among the responders, suggesting that dual B and T cell inhibition can provide durable remissions with a short course of therapy (144). Successful use of cyclosporin post rituximab has been described in case report and case series (141, 142). In a recent meta-analysis cyclosporine-based combinations improved ORR and CR rates while reducing the rate of relapse (145). Importantly, this improvement was achieved without increasing the rate of adverse events. These findings suggest that cyclosporine alone or in combination with other agents can provide deeper and more durable responses in patients with refractory ITP.

### 5.8 Platelet desialylation

Multi-refractory ITP patients have a higher proportion of anti-GPIb/IX antibodies, which can cause platelet desialylation, leading to accelerated clearance of platelets via the hepatic AMR (18) and an elevated number of desialylated platelets (21).

Oseltamivir, is an inhibitor of neuraminidase, the enzyme involved in the desialylation of platelets. In a prospective case series of seven patients with persistent, chronic, or refractory ITP treated with oral oseltamivir 75 mg twice daily for 5 days initial responses were seen in all patients but were not sustained (146). In an open-label randomized phase 2 trial in newly diagnosed ITP addition of oseltamivir (75 mg twice a day for 10 days) to dexamethasone (40 mg/day for 4 days) produced significantly higher 6-month sustained response rate (53 vs. 30%; OR 2.17; P = 0.032). Interestingly, patients with anti-GPIb/IX did not achieve better responses with oseltamivir (147).

### 5.9 Thrombopoietin receptor agonists

Thrombopoietin receptor agonists (TPO-RA) interact with the thrombopoietin (TPO) receptor, induce a conformational change, triggering activation of the JAK2/STAT5 pathway, enhancing proliferation of megakaryocyte progenitors which leads to increased platelet production (148). TPO-RAs have been successfully used in ITP since 2006 (149).

Currently there are 3 TPO-RAs including romiplostim, eltrombopag, avatrombopag approved by FDA based on several prospective randomized phase 3 clinical trials with response seen in 60–90% of the cases, including rituximab refractory patients.

Due to their favorable safety profile TPO-RAs have increasingly been used in second line setting. Thrombosis is a complication seen in 6% cases and while previously a concern, bone marrow fibrosis reported in 1.4 to 6% of cases, is reversible on discontinuation of the agent. Eltrombopag has dietary restrictions and risk of hepatotoxicity, while most recently approved avatrombopag can be administered in patients with hepatic dysfunction (150–154). Romiplostim is subcutaneously administered peptibody that directly binds to the TPO binding site in a competitive manner, whereas eltrombopag and avatrombopag are oral small molecules that binds to a trans-membrane site. Eltrombopag also exhibits off-target effects, acting as a chelator of both extra- and intra-cellular calcium and iron, and facilitating the transport of iron out of cells. This iron-chelating property of eltrombopag results in a TPO-independent stimulation of stem cells and megakaryocyte precursors (155). Lusutrombopag is currently approved for the treatment of thrombocytopenia in patients with chronic liver disease (156). However, a study evaluating its effectiveness in treating ITP was terminated prematurely due to the inability to achieve the study objectives (NCT01054443). Hetrombopag is a TPO-RA approved in China with similar response rate in patients with relapsed or refractory ITP (157).

Head-to-head comparison between TPO-Ras are lacking. Options of the agent should take into consideration the way of administration (158). Switching between TPO-Ras can be considered based on lack of efficacy, platelet fluctuations, safety, and tolerability. If switching was due to lack of efficacy, the response rate to the second TPO-RA could be still as high as 65%. Almost every patient switched TPO-RA due to reasons other than lack of efficacy continued to respond after the switch (93%) (159). In a recent multicenter observational study of 44 patients with chronic ITP who had not responded or were intolerant to either romiplostim or eltrombopag, 41 patients (93%) achieved a platelet response (platelet count of ≥ 50 × 109/L) after switching to avatrombopag (160).

Due to their role as growth factors and the observation that most patients experienced relapse soon after discontinuing TPO-RA treatment (148, 153). TPO-RAs were traditionally viewed as chronic supportive therapy with no effect on the immune system. However, retrospective studies have indicated that 15-20% of patients may achieve long term remissions off therapy, suggesting that these agents may also have an immunomodulatory effect in ITP (161). Restoration of Tregs levels and function in peripheral blood and spleen has been proposed as a potential mechanism (162). In a prospective multicenter study of 48 patients with persistent or chronic ITP and complete response to TPO-RA therapy, sustained response off-treatment and sustained complete response off-treatment were achieved in 56% and 31% of patients at week 24 and 52% and 29% at week 52, respectively, suggesting that TPO-RA discontinuation can be considered in ITP patients in long-term CR (163).

### 5.10 Complement inhibition

Complement plays an important role in ITP pathogenesis, with classical complement pathway activated by antibodies binding to platelets leading to complement-dependent cytotoxicity (CDC) as well as phagocytosis by macrophages which recognize C3 via complement receptor 1 (CR1) (13–15).

In an vitro study increased complement activation was demonstrated in 47% patients with ITP with increased C1q deposition in 42% of patients and was inhibited by TNT003, a murine monoclonal antibody that targets C1s and inhibits classical complement pathway (164).

Sutimlimab, a humanized C1s IgG4 monoclonal antibody, was evaluated in a Phase I trial in 12 patients with chronic severe ITP who failed at least two prior therapies, including 8 patients with insufficient response to rituximab and administered as an infusion Days 0 and 7, then every 2 weeks for up to 21 weeks with an option to enter a long term extension after 9 week washout (165). Durable overall response (platelet ≥50 G/L in ≥50% of follow-up visits) was seen in 42% of patients, with 33% of patients achieving CR (platelet count ≥100 G/L) with the median time to response of 2 days. The median platelet count returned to baseline during the washout, suggesting need for ongoing therapy to maintain response. Complement inhibition is a promising therapeutic target in a subset of patients with complement activation which underscores the importance of identifying biomarkers that can help determine which patients will respond to treatment. A phase 2 study evaluating BIVV020, a C1s inhibitor which can be self-administered subcutaneously, has been recently completed but results are not yet available (NCT04669600). Inhibition of the alternative complement pathway with iptacopan, a Factor B inhibitor is currently being evaluated in a phase 2 basket study in ITP and cold agglutinin disease (NCT05086744).

## 6 Conclusion

Most adult patients with ITP relapse after initial therapy and require subsequent treatment. Rituximab therapy has been utilized in relapsed and refractory ITP with high response rates with short treatment duration. However, remission duration is limited, and many patients become refractory to therapy. In addition, immunosuppressive effect and impairment of response to vaccination has become a concern, particularly in the era of COVID-19 pandemic. There is an unmet need for novel therapeutic approaches that are safe, tolerable and can overcome rituximab resistance. Potential escape mechanisms include expansion of CD19+ B cells, T cells, plasma cells and complement. Based on the mechanism, several available approaches are proposed to overcome rituximab resistance. Surgical intervention splenectomy after rituximab is more effective than rituximab alone. Novel agents that interfere with FcγR dependent phagocytosis including fostamatinib, rilzabrutinib and inhibition of FcRn mediated pathogenic antibody recycling, and complement inhibitors are demonstrating their efficacy when patients relapse after rituximab. Plasma cell directed therapies and BAFF inhibitors could be used either simultaneously in addition to rituximab to provide synergy or solely after rituximab. Last but not least, conventional immune-suppressant agents which target T cells should also be considered given the potential synergistic effect of B and T cell depletion. TPO-Ra’s remain an attractive therapy with potential of treatment free remission after a stable response.

## Author contributions

All authors listed have made a substantial, direct and intellectual contribution to the work, and approved it for publication.
